# Toward a Neurobiological Model of Gestalt Confluence: Thalamocortical Integration as a Hypothetical Framework for Contact Interruption

**DOI:** 10.3390/brainsci16060598

**Published:** 2026-05-31

**Authors:** Enrica Tortora, Valeria Cioffi, Chiara Scognamiglio, Lucia Luciana Mosca, Enrico Moretto, Roberta Stanzione, Francesco Marino, Giovanni Salonia, Claudia Montanari, Oliviero Rossi, Claudio Billi, Alexander Lommatzsch, Antonio Ferrara, Stefano Crispino, Elena Gigante, Mariano Pizzimenti, Roberta Melis, Efisio Temporin, Raffaele Sperandeo

**Affiliations:** 1 SiPGI-Scuola Post-Laurea di Psicoterapia della Gestalt Integrata, 80058 Torre Annunziata, Italy; dr.valeria.cioffi@gmail.com (V.C.);; 2 Istituto di Terapia della Gestalt, H.C.C. (Centro di Comunicazione Umana), KAIROS, Via Virgilio, 10, 97100 Ragusa, Italy; 3 ASPIC Scuola di Psicoterapia, Via Vittore Carpaccio, 32, 00147 Roma, Italy; 4 IPGE Istituto di Psicoterapia della Gestalt Espressiva, Via Costantino Morin, 24, 00195 Roma, Italy; 5 Scuola di Specializzazione in Psicoterapia della Gestalt CGV, Centro Gestalt Viva Claudio Naranjo, Via Leonardo Cambini 44, 57125 Livorno, Italy; 6 IGP Istituto Gestalt di Puglia, Via De Simone, 29, 73010 Arnesano, Italy; 7 IGAT Istituto di Psicoterapia della Gestalt e Analisi Transazionale, Via Pirro Ligorio, 20, 80129 Napoli, Italy; 8 IGA Istituto Gestalt Analitica, Via Padre Semeria, 33, 00154 Roma, Italy; 9 SIPGI Scuola in Psicoterapia Gestaltica Integrata, Via Osorio, 24, 91100 Trapani, Italy; 10 SGT Scuola Gestalt Torino, Via Po, 14, 10123 Torino, Italy; 11 SinaPsi Istituto di Psicoterapia della Gestalt, Via Garavetti, 22, 09129 Cagliari, Italy; 12 IGR Istituto Gestalt Romagna Via Cesarea, 88, 48121 Ravenna, Italy; 13 Department of Neuroscience and Reproductive and Odontostomatological Sciences, University of Naples “Federico II”, 80131 Naples, Italy

**Keywords:** Gestalt therapy, confluence, thalamocortical integration, salience network, figure-ground differentiation, interoception

## Abstract

**Highlights:**

**What are the main findings?**

- Dysfunctional confluence, a core mechanism of contact disruption in Gestalt therapy, may be theoretically reframed in neurobiological terms as a possible failure of the thalamocortical integrative axis to complete the transition from globally distributed somatosensory activation to the formation of differentiated figures within the salience network.
- The three phenomenological dimensions of confluence identified by Cioffi et al. (2025)—blurred boundaries, undifferentiation, and avoidance of contact—appear traceable to distinct yet convergent disruptions of thalamic filtering, thalamocortical synchronization, and amygdala-mediated suppression of salience-relevant signals, respectively.

**What are the implications of the main findings?**

- The proposed framework suggests reconsidering somatic desensitization not merely as a symptom of dysfunctional confluence but as a potential neurobiological maintenance mechanism, raising the possibility that body-based therapeutic interventions may operate at the level of thalamocortical signal quality and not exclusively at the phenomenological level.
- By translating Gestalt clinical constructs into terms consistent with established affective neuroscience, the model aims to generate testable hypotheses—including neuroimaging studies of thalamocortical connectivity and psychophysiological paradigms on interoceptive sensitivity—that may open a productive dialogue between humanistic psychotherapy and neuroscientific investigation.

**Abstract:**

**Background/Objectives:** The integration of psychotherapeutic theory with contemporary neuroscience represents one of the most productive frontiers of clinical research. The fundamental theoretical constructs of Gestalt therapy have been developed with considerable clinical depth, yet their neurobiological foundations remain largely unexplored. Confluence, one of the most debated mechanisms of contact disruption in Gestalt therapy, has recently been described as characterized by a three-dimensional conceptual structure: blurred boundaries, undifferentiation, and avoidance of contact. **Methods**: In this hypothesis article, we start from this structure and from neuroscientific evidence on thalamic filtering, thalamocortical synchronization, and salience attribution. **Results:** We propose an original theoretical framework—explicitly hypothesis-generating rather than empirically validated—for dysfunctional confluence, understood as a putative disruption of the transition from globally distributed somatosensory activation to the formation of differentiated figures in the salience network. **Conclusions:** The proposed correspondences are intended as heuristic mappings aimed at generating testable hypotheses and opening a productive dialogue between Gestalt theory, affective neuroscience, and clinical practice.

## 1. Introduction

The integration of psychotherapeutic theory with contemporary neuroscience represents one of the most challenging and productive frontiers of clinical research. Gestalt therapy, one of the most influential humanistic-experiential approaches, is no exception: its core theoretical constructs—the contact cycle, field theory, and relational boundary processes—have been developed with considerable clinical depth, yet their neurobiological foundations remain largely unexplored [1,2]. This gap limits not only the scientific validation of Gestalt therapy but also its ability to contribute to the broader neuroscientific literature on perception, self-regulation, and interpersonal processes.

A recent systematic semantic analysis of confluence within the Italian Gestalt therapy community [3] represents a significant step toward addressing this gap. Drawing on the theoretical contributions of senior trainers and directors representing the majority of Italian Gestalt training programs, the study identified a shared three-dimensional conceptual structure of confluence: blurred boundaries, undifferentiation, and avoidance of contact. Confluence emerged as a contextual mechanism within the contact/experience process, existing along a functional-dysfunctional continuum determined by intensity, phase of occurrence, and temporal persistence. Despite achieving solid conceptual clarity and inter-institutional integration, the study identified a critical limitation: the absence of an explicit neurobiological framework capable of explaining the mechanism of confluence. The present article aims to fill this neurobiological gap, building upon the three-dimensional structure identified by Cioffi et al. (2025) [3]. It should be noted from the outset that the model proposed here is a theoretical, hypothesis-generating framework, not an empirically validated account; the epistemological scope of this proposal is discussed in detail in Section 7.1.

## 2. Aim

Drawing on established neuroscientific literature on the thalamus as a multimodal integrative hub and the salience network as a figure-ground differentiation system [4,5,6], we propose a neurobiological framework for dysfunctional confluence, understood as a disruption of the thalamocortical integrative axis. The three phenomenological dimensions identified by Cioffi et al. (2025) [3]—blurred boundaries, undifferentiation, and avoidance of contact—are traced back to a common neurobiological denominator, identifying potential targets for future empirical investigations. This framework aims to link Gestalt field theory with affective neuroscience, offering a theoretically grounded model that can inspire both empirical research and clinical practice in Gestalt psychotherapy.

## 3. Materials and Methods

This contribution takes the form of a hypothesis article drawing on a selective review of neuroscientific literature, aimed at identifying evidence consistent with the clinical constructs of Gestalt therapy, without claiming systematic exhaustiveness. The literature search was conducted in the Google Scholar, PubMed, and PsycINFO databases using the following terms: thalamus, salience network, thalamocortical integration, interoception, figure-ground, Gestalt therapy, confluence, contact cycle, with priority given to peer-reviewed publications from the last twenty years. The correspondences between Gestalt constructs and neuroscientific data are the result of a two-phase conceptual integration process: the analysis of the neurobiological functional requirements involved in the three phenomenological dimensions of confluence, followed by the identification of anatomical-functional structures consistent with these requirements. The resulting mappings do not constitute empirical confirmations, but rather heuristic tools for generating testable hypotheses, as explained in Section 7.1.

## 4. Conceptual Context: The Three-Dimensional Structure of Confluence

### 4.1. Confluence in Gestalt Therapy: Classical Foundations

**Figure 1:**
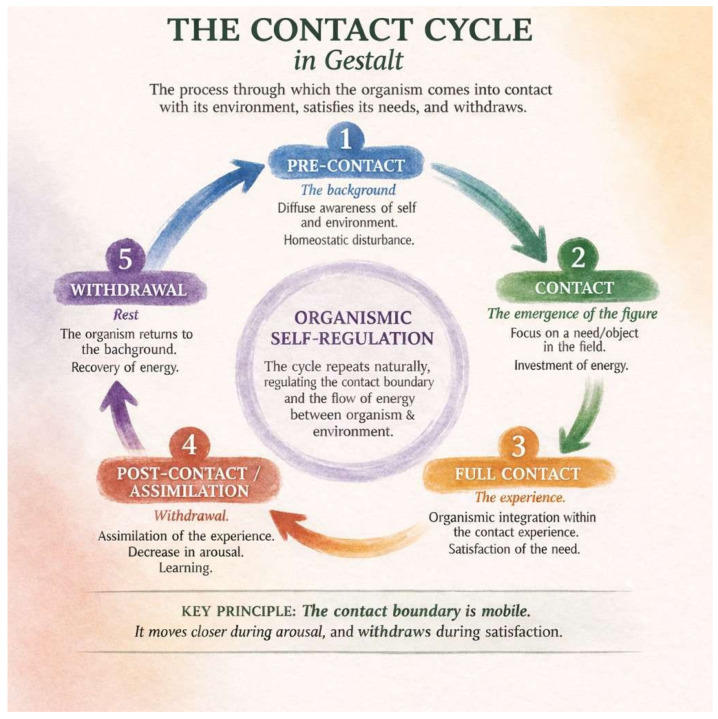
The Contact Cycle in Gestalt Therapy. A representation of the stages of the organismic self-regulation process, through which the organism moves from an undifferentiated experiential background to the emergence of a salient figure, and on to its completion and subsequent withdrawal.

Confluence is one of the most debated mechanisms for interrupting the contact process in Gestalt therapy, first introduced in the seminal work by Perls, Hefferline, and Goodman (1951) [7]. In the original formulation, confluence refers to a condition in which the boundary between the self and the environment becomes absent or indistinct, preventing the organism from recognizing disturbances in homeostatic equilibrium and thus blocking the emergence of new gestalts. The contact cycle (Figure 1) provides the broader framework within which confluence operates and is closely related to the concept of the field of experience, that is, the set of interactions between the organism and the environment. Through this cycle, the organism, in continuous interaction with the environment, organizes sensory and perceptual information into a unified perceptual experience, called a “Gestalt.” Figure 1 illustrates how the organism moves from an undifferentiated experiential background toward the emergence of a salient figure that guides homeostatic rebalancing and conscious experience [8,9]. Confluence, in its dysfunctional form, interrupts this process at its origin: it blocks the transition from the pre-contact phase, preventing the organism from forming a differentiated figure-ground structure and halting the entire experiential cycle [7,10]. As described by Perls et al., confluence is pathological only when sustained as a means to prevent contact; when it occurs at the end of the contact cycle, however, it facilitates the organism’s assimilation and growth. This dual nature—functional at the boundaries of the contact cycle, dysfunctional when persistent and rigid—has been a central theoretical tension, addressed with limited consensus by subsequent authors [11,12,13].

### 4.2. The Three-Dimensional Structure: Results of the Semantic Analysis

**Figure 2:**
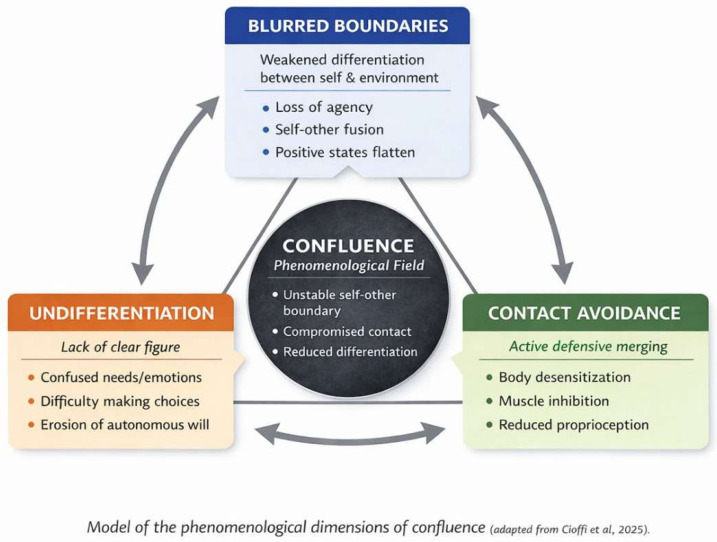
Model of the three phenomenological dimensions of confluence (adapted from [3]). Confluence is conceptualized as a phenomenological field comprising three interconnected dimensions: (1) blurred boundaries, characterized by reduced differentiation between the self and the environment; (2) undifferentiation, in which the figure-ground formation is compromised; and (3) avoidance of contact, understood as an active defense mechanism. The three dimensions do not represent discrete categories, but rather dynamic processes that coexist and influence one another within the experience.

The systematic semantic analysis conducted by Cioffi et al. (2025) [3] in eight Italian Gestalt training institutes affiliated with FISIG identified a shared three-dimensional conceptual core of confluence, confirmed by inter-rater agreement (87.5% agreement) and inter-rater reliability (Cohen’s κ = 0.87). Table 1 and Figure 2 illustrate the three dimensions—blurred boundaries, undifferentiation, and avoidance of contact—as distinct but interrelated aspects of the same underlying phenomenon.

The dimension of blurred boundaries emerged as the most frequently identified characteristic, present in seven out of eight contributions. It describes a weakened differentiation between the self and the environment: an indistinct or absent experience of where the self ends and the other begins. This dimension encompasses a broad phenomenological range, from positively valenced experiences of fusion—such as orgasm, creative flow, or early infantile bonding—to pathological forms involving loss of agency and psychotic fusion between the self and the other. The functional expression of blurred boundaries is characteristic of the early stages of self-development and the assimilative endpoints of the contact cycle; its dysfunctional persistence generates a progressive attenuation of the sense of personal identity within the organism-environment field.

Undifferentiation was identified in six out of eight contributions and describes the inability to distinguish one’s own sensations, emotions, and evaluations from those of others. In Gestalt terms, this reflects a persistence in the pre-contact d-phase, in which sensory information remains globally organized and unstructured, preventing the formation of a clear gestalt. Clinically, undifferentiation manifests as confusion regarding one’s own needs, desires, and feelings; an inability to extract clear figures from the experiential context; and, in its most severe expressions, a progressive erosion of autonomous will and decision-making capacity [14].

Contact avoidance was present in five out of eight contributions and represents an active defensive mechanism rather than a passive absence of contact. This dimension captures the organism’s withdrawal into a protective fusion in order to evade the vulnerability inherent in authentic differentiation and encounter. Unlike the other two dimensions, avoidance of contact captures the teleological quality of confluence, namely its function of protection against differentiation anxiety and potential rejection. The bodily manifestations associated with this dimension include desensitization, functional muscle paralysis, and reduced somatic sensitivity [15,16].

### 4.3. The Functional-Dysfunctional Continuum

It is important to emphasize that semantic analysis has also revealed an early neurobiological insight within the Italian Gestalt community: several authors have implicitly referred to embodied phenomena—somatic desensitization, muscular inhibition, perceptual blurring—suggesting that confluence has a putative biological correlate not yet formally theorized. This observation directly motivates the neurobiological model proposed in this article.

The three phenomenological dimensions of confluence converge toward a common neurobiological denominator: an alteration of the process through which the organism constructs, starting from heterogeneous sensory signals, a unified and differentiated perceptual experience—the inaugural moment of the contact cycle in which the undifferentiated background gives way to the salient figure. In dysfunctional confluence, this moment does not occur: the figure does not emerge, the Gestalt does not form, and the experiential cycle remains stuck at its origin. Identifying the hypothesized neurobiological correlate of this failure requires pinpointing a structure that integrates, filters, and synchronizes heterogeneous multisensory signals into a coherent, action-oriented experiential output—a structure that, as argued in the following sections, finds its prime anatomical and functional candidate in the thalamus.

## 5. The Thalamus as a Multimodal Integrative Center: From Sensory Signals to Gestalt Formation

### 5.1. Thalamic Architecture and Integrative Functions

The thalamus occupies a unique position in the neuroanatomy of conscious experience, making it particularly relevant to the model proposed here. Located at the geometric center of the brain, above the midbrain, it constitutes the primary precortical integrative center through which virtually all sensory and perceptual information—with the exception of olfactory inputs—converges before reaching cortical areas [17,18]. This crossroads position—between ascending signals from the body and descending cortical modulation—makes the thalamus the natural neurobiological candidate for the Gestalt formation process described in the previous section. By gathering sensory tracts, motor tracts, and visceral signals, the thalamus is not a passive relay station, but rather an active processor that filters and synchronizes this information to produce a unified subjective experience. Neuropsychological research has shown that the thalamus possesses intrinsic integrative capabilities beyond simple data transfer: together with the brainstem and hypothalamus, it can support conscious representations of pain, itching, and temperature even when primary cortical areas such as the bilateral insula are damaged [19].

The first is a filtering function, mediated by the thalamic reticular nucleus (TRN), a thin layer of GABAergic fusiform neurons that wraps laterally around the dorsal thalamus and receives afferents from the cerebral cortex and other thalamic nuclei. TRN neurons extend inhibitory dendrites into thalamic nuclei over which they exert inhibitory control: they selectively suppress distracting inputs and maintain attention on stimuli relevant to homeostatic rebalancing [5,20]. It is crucial that the TRN is also innervated by the amygdala, providing a mechanism for emotion-driven attentional shifts involved in the defensive dynamics of contact avoidance [21]. Zikopoulos and Barbas (2012) [21] demonstrated in primates that this amygdala → TRN pathway forms synapses unusually close to TRN cell bodies, with larger and more efficient terminals than those from the orbitofrontal cortex or the mediodorsal thalamic nucleus—a structural feature suggesting that amygdalar input exerts a particularly rapid and powerful inhibitory influence over thalamic signal gating. We hypothesize that in individuals with dysfunctional confluence, chronically elevated amygdala activity—driven by the implicit threat associated with self-other differentiation—tonically suppresses TRN-mediated signal amplification, preventing salience-relevant interoceptive and proprioceptive signals from reaching the coherence threshold necessary for figure formation in the salience network.

The second is a synchronization function, through which the thalamus coordinates the simultaneous activity of primary and associative cortices, producing a unified experiential output. This function is particularly evident in the pulvinar, which generates multimodal synchronization between auditory and visual processes, and in the diffuse projection nuclei of the central zone—the centromedial (CM), centrolateral (CL), and parafascicular (PF) nuclei, which regulate states of alertness, adaptive behavioral responses, pain responses, and behavioral flexibility [6,22,23].

The mediodorsal nucleus (MD), which is strongly interconnected with the prefrontal cortex and the limbic system, plays an integrative role between emotional signals and higher-order decision-making and evaluative processes, making it particularly relevant to contact-avoidance dynamics in which emotional regulation interferes with figure-ground differentiation [24,25].

These two functions of the thalamus appear to represent the putative neurobiological counterpart of the integrative process of forming a unified perceptual gestalt described by GT. In Gestalt terms, thalamic filtering corresponds to the selection of what emerges as a relevant figure against an undifferentiated background, while synchronization corresponds to the very act of perceptual unification that confers coherence and subjective salience to the emerging experience. It is precisely in this dual operation—selecting and unifying—that the thalamus functions as the proposed neurobiological correlate of the pre-reflective moment in which the organism begins to differentiate itself from the field.

### 5.2. Sensory Inputs and the Neuromodulatory Context

Sensory information reaches the thalamus via two main ascending systems. The lemniscal pathway transmits detailed, topographically organized mechanoproprioceptive information—the hypothesized neurobiological basis of what Gestalt therapy refers to as proprioceptive awareness in the contact cycle. The spinothalamic pathway, on the other hand, carries information regarding thermal, pain, and visceral sensitivity, including the emotional and affective valence of contact, making it particularly relevant to the intersubjective dimensions of the organism-environment field [26,27]. Alongside these signals, visual and auditory inputs converge on the thalamus via the geniculate nuclei. It is from this multisensory synthesis that the organism first acquires a proto-conscious sense of itself as a differentiated entity within an environment [28,29]. This integrative process, however, operates within broader neuromodulatory conditions. It is embedded in a larger ascending neuromodulatory system, the ascending reticular activating system (ARAS), which modulates the organism’s arousal state and thus determines the basic conditions under which thalamic filtering and synchronization operate. The ARAS comprises a dorsal component characterized by glutamatergic and cholinergic activity associated with attentive vigilance, and a ventral component involving dopaminergic, noradrenergic, and serotonergic activity that promotes motor initiative, reward mechanisms, and regulation of the sleep–wake cycle [30].

In summary, the ARAS provides the neurophysiological “background” (arousal and vigilance) upon which the thalamus can operate to bring forth the experiential “figure” through its filtering and synchronization processes. This dual architecture—topographically precise transmission and globally distributed activation—will constitute the anatomical basis of the neurobiological model proposed in Section 7.

## 6. The Salience Network and the Proposed Neurobiological Basis of Figure-Ground Formation

### 6.1. The Concept of Salience and Its Relevance to Gestalt Theory

The concept of salience refers to a stimulus’s ability to capture attentional resources and guide adaptive behavioral responses. Fundamentally, a stimulus’s salience is not determined exclusively by its physical properties, but primarily by its relevance to the organism’s homeostatic state and the subjective motivational context in which it occurs [31,32]. The attribution of salience is shaped bidirectionally: top-down via cortical attentional control and bottom-up via interoception, proprioception, and emotional states. In GT, the emergence of a figure from the experiential background is not a passive perceptual event, but an active and motivated process, determined by the organism’s homeostatic needs, its affective state, and the available environmental affordances [7,8]. The salience network controls attention and guides adaptive behavior in response to homeostatic disruptions [4,32]. We propose that this architecture is the functional counterpart of what Gestalt theory calls motivated figure-ground differentiation: the organism’s active engagement with those aspects of the field relevant to its current needs.

### 6.2. Anatomical Structure of the Salience Network

The salience network (SN), first comprehensively described by Seeley (2019) [4] and also known as the cingulo-opercular network [33], comprises a set of anatomically and functionally connected cortical and subcortical regions that cooperate in the identification and prioritization of salient stimuli. Its main nodes are as follows.

The dorsal anterior cingulate cortex (dACC) receives somatosensory afferents related to visceral organs and contributes to emotional processing, empathy, the experience of physical and social pain, and the modulation of sympathetic responses, including heart rate and general arousal [34]. In Gestalt terms, the dACC corresponds to the affective-evaluative dimension of the contact boundary, that is, the capacity to register the emotional significance of the organism-environment encounter.

The anterior insula and the orbitofrontal cortex are centrally involved in interoception—the conscious monitoring of the organism’s internal physiological state—as well as in the awareness of one’s own emotional states and those of others, and in the perception of emotional pain [28,35]. The anterior insula occupies a particularly strategic position in the model proposed here, as it constitutes the primary cortical interface between interoceptive signals and conscious experience—the neural site where bodily signals are transformed into perceived sensation.

The temporo-parietal junction (TPJ) and parts of the middle and superior temporal gyri are involved in directing attention toward external stimuli and in distinguishing between self-generated and externally generated signals, a function directly relevant to the boundary processes between self and other described in Gestalt theory as the basis of authentic contact [36].

The amygdala and hypothalamus contribute to the processing of emotional states and the vegetative and endocrine modulation of emotions, providing the SN with its connection to the body’s hormonal and autonomic regulatory systems.

### 6.3. Thalamic Connections of the Salience Network

Functional neuroimaging studies converge in suggesting that the thalamus plays a significant role within the salience system, participating in the detection of homeostatic disturbances and the orientation of neural resources toward relevant stimuli [37,38]. This role appears to be mediated by a group of nonspecific thalamic nuclei with diffuse projections to limbic cortical areas, the hypothalamus, and the striatum. The irregular axonal arboreations of these nuclei allow for the simultaneous activation of distant cortical regions involved in homeostatic rebalancing [39,40].

The paraventricular (PV) nucleus of the thalamus serves as a particularly informative model for the generation of thalamic salience. The PV receives excitatory input from brainstem nuclei, including the locus coeruleus and the reticular formation, and projects to the prefrontal cortex (PFC), the nucleus accumbens (NAc), the bed nucleus of the stria terminalis (BNST), and the amygdala—a circuit that integrates homeostatic signals, reward processing, and threat assessment [41,42]. Of particular interest to the model proposed here, single-unit recordings have shown that neurons in the posterior PV can be activated by both rewarding and aversive stimuli, suggesting that the PV encodes stimulus salience independently of valence [43]. This valence-independent encoding of salience is directly relevant to the Gestalt concept of the figure as that which is most relevant for homeostatic rebalancing, regardless of whether the relevance is appetitive or aversive.

### 6.4. From the Salience Network to the Contact Cycle: A Parallel Architecture

Table 2 summarizes the proposed correspondences between the phases of the Gestalt contact cycle and the neurobiological correlates hypothesized within the thalamic-salience network system.

**Figure 3:**
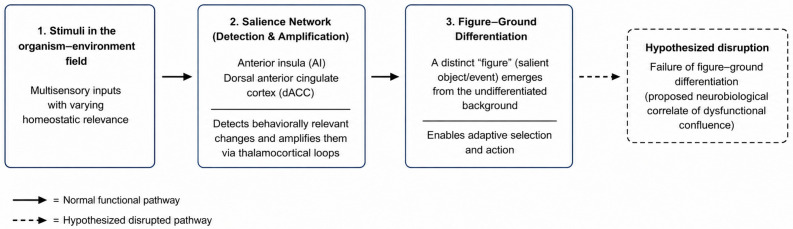
Thalamocortical pathway of figure-ground formation. The salience network identifies and amplifies homeostatically relevant stimuli, enabling the emergence of a distinct figure from the undifferentiated experiential background. Disruption of this process is proposed as a neurobiological correlate of dysfunctional confluence.

The pre-contact sensory phase of the Gestalt cycle, in which the organism’s bodily state constitutes the undifferentiated background, corresponds neurobiologically to the activation of somatosensory networks and ascending thalamic pathways, through which proprioceptive and interoceptive signals generate a proto-conscious and pre-verbal sense of self [26,44]. This is a globally distributed state devoid of figures: the organism is present and aroused, but no specific figure has yet emerged from the background. In the pre-contact perceptual phase, a specific salient figure emerges from the background, bringing with it the awareness of the homeostatic disturbance. This phase corresponds neurobiologically to the activation of the salience network, which identifies the homeostatically most relevant stimulus in the organism-environment field, amplifies it against the background of diffuse thalamic activation, and makes it available to higher-order executive and evaluative processes. This is the neurobiological moment of figure formation: the emergence of a clear gestalt from the undifferentiated background (Figure 3).

The interruption of this process could lead to a failure of figure-background differentiation, which we propose may constitute a plausible neurobiological correlate of dysfunctional confluence. The epistemological status and theoretical scope of this proposal are discussed in Section 7.

## 7. Confluence as a Thalamocortical Integrative Dysfunction: A Neurobiological Model

### 7.1. Epistemological Premises and Scope of the Proposal

The neurobiological architecture described in Section 5 and Section 6 provides the conceptual infrastructure for the interpretive framework proposed in this manuscript. Before presenting this framework, however, an epistemological clarification is necessary. The model we propose is a theoretical hypothesis, not an empirically validated account. To date, no electrophysiological or neuroimaging study has directly measured confluence as a neurobiological variable, and no existing literature has investigated the neural correlates of the three dimensions identified by Cioffi et al. (2025) [3]. The correspondences proposed below are therefore heuristic mappings—theoretically coherent extrapolations from established neuroscientific findings to a clinical construct that has not yet been subjected to neuroscientific investigation. Their value lies not in their empirical confirmation—which awaits future research—but in their ability to generate testable hypotheses and to link two bodies of knowledge—Gestalt clinical theory and affective neuroscience—that have developed in near-complete isolation from one another. It should also be noted that confluence and thalamocortical integration belong to different levels of description—phenomenological-clinical and neurobiological, respectively—and the mappings proposed here are correspondences between these levels, not ontological reductions.

With this clarification, we propose that dysfunctional confluence can be understood as an interruption of the thalamocortical integrative process—specifically, a failure of the transition from the pre-contact sensory phase, characterized by globally distributed somatosensory activation, to the pre-contact perceptual phase, in which salience network engagement produces a differentiated figure.

### 7.2. The Thalamocortical Axis as the Hypothesized Neurobiological Correlate of Confluence

The neurobiological architecture described in Section 5 and Section 6 converges into a single anatomical-functional system: the thalamocortical axis. This is not a simple relay pathway, but a bidirectional circuit in which the thalamus coordinates ascending sensory signals, the reticular network filters them by homeostatic relevance, and the cortex returns descending modulation via the salience network. It is precisely this feedforward/feedback loop that allows the system to transform diffuse somatosensory activation into a unified and oriented perceptual experience: the thalamus detects and directs neural resources toward homeostatically relevant stimuli, while the cortex—in particular the anterior insula and anterior cingulate cortices—processes their emotional and subjective meaning, producing the synchronization of primary and associative cortices from which the Gestalt emerges as a unified and global experience [4,45]. The theoretical plausibility of this mapping rests on a functional equivalence: both dysfunctional confluence and the proposed thalamocortical failure describe a state in which the organism remains globally activated but perceptually undifferentiated—present in the field, yet unable to extract a figure from it [4,5,6,26,45].

Under conditions of normal thalamocortical functioning, a homeostatic disturbance triggers a sequence of neurobiological events that, at the macroscopic level of subjective experience, corresponds to the pre-contact Gestalt cycle. Ascending somatosensory signals—conveying interoceptive and proprioceptive information via the lemniscal and spinothalamic pathways—are integrated at the thalamic level into a proto-conscious representation of the current homeostatic condition. This globally distributed, pre-verbal representation constitutes the neurobiological correlate of the pre-contact sensory phase: the body as a background, the site from which a figure has not yet emerged. Thalamic filtering by the TRN, modulated by ARAS-induced excitation and the emotional weighting provided by amygdala afferents, allows for the progressive amplification of homeostatically relevant signals. When this amplification reaches a sufficient degree of spatial and temporal coherence, the salience network is activated: the anterior insula, the dorsal anterior cingulate cortex, and associated nodes identify the most relevant disturbance in the organism-environment field and bring it forth as a salient figure against the now-organized background. This is the neurobiological correlate of the pre-contact perceptual phase, the moment when a specific and differentiated gestalt crystallizes from the undifferentiated field [4,45].

**Figure 4:**
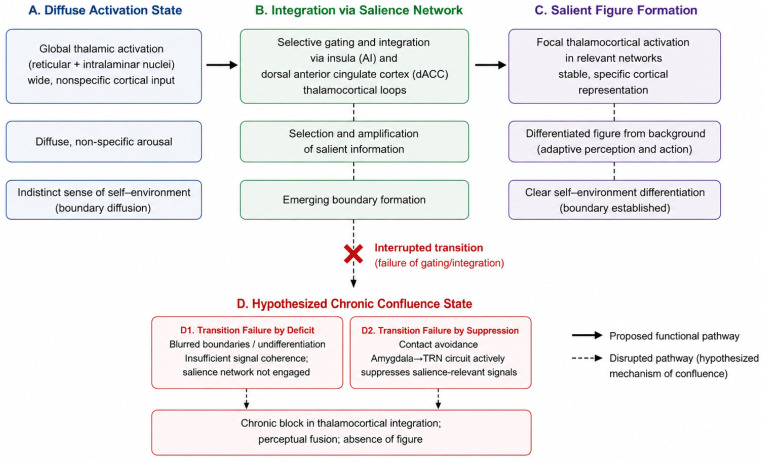
Confluence as a thalamocortical integrative dysfunction: a neurobiological model. The figure illustrates the blockage of the transition between the state of diffuse activation (**left**)—characterized by global thalamic activation and an indistinct sense of self—and the formation of the salient figure in the salience network (**right**), mediated by the insula and dorsal anterior cingulate cortex (dACC). In dysfunctional confluence (**lower center**), this transition is interrupted through two distinct mechanisms: signal deficit (blurred boundaries/undifferentiation) or active suppression via the amygdala → TRN circuit (contact avoidance), both resulting in perceptual fusion and a chronic block in thalamocortical integration.

We propose that dysfunctional confluence represents a failure of this sequence, specifically a chronic inability to complete the transition from globally distributed somatosensory activation to the formation of differentiated figures in the salience network (Figure 4). The organism remains stuck in a state analogous to the pre-contact sensory phase: homeostatically activated but perceptually undifferentiated—present in the field, yet unable to extract a figure from it or engage in adaptive contact. A partial exception to this convergence toward a common denominator concerns the dimension of contact avoidance, which is phenomenologically distinct from the other two due to its teleological and actively defensive character: while blurred boundaries and undifferentiation describe a state of differentiative deficit—the absence of a clear figure against the background—contact avoidance captures a process of active suppression, whose hypothesized neurobiological correlate lies in the amygdala-TRN inhibitory circuit, through which the very prospect of differentiation can trigger a defensive response that selectively suppresses thalamic signals relevant to salience even before they reach the threshold necessary for figure formation. In both cases the outcome is the same—chronic inability to complete the transition to a differentiated gestalt—but the mechanism differs: deficit of amplification versus active suppression.

### 7.3. The Functional-Dysfunctional Continuum: A Neurobiological Interpretation

The proposed framework must, however, account for a distinctive feature of the construct, already identified by Cioffi et al. (2025) [3]: its functional-dysfunctional continuum (Table 3). Functional confluence—situated at the boundaries of the contact cycle, allowing for early attachment, creative immersion, and organismic assimilation—is not an absence of thalamocortical integration, but a particular mode of it: characterized by the temporary and contextually appropriate loosening of the signal’s degree of differentiation. The putative neurobiological basis of this functional state may overlap substantially with that of dysfunctional confluence; the difference lies not in the mechanisms involved, but in their temporal regulation, contextual modulation, and degree of reversibility.

This interpretation is consistent with contemporary neuroscientific models that describe neurobiological processes at the micro-anatomical level as the putative generative basis of psychological processes at the relational level [45,46,47]. Functional confluence may represent the normal functioning of the thalamocortical integrative system under conditions of reduced homeostatic disturbance or post-contact assimilation: states in which the precise delineation of boundaries between self and environment is temporarily less urgent on a functional level. Dysfunctional confluence, on the contrary, represents the solidification of this state into a temporally persistent and context-independent pattern, which chronically prevents the emergence of a differentiated gestalt and disrupts the organism’s capacity for adaptive contact with its environment.

## 8. Discussion

### 8.1. The Body and the Therapeutic Relationship as a Neurobiological Access Point

The proposed framework does not reduce confluence to its neural correlates but offers a descriptive register complementary to the phenomenological and relational ones. If dysfunctional confluence involves an interruption of thalamocortical integration at the level of interoceptive and proprioceptive synthesis, then somatic self-awareness becomes a primary diagnostic and therapeutic indicator of confluent functioning. This is not new to Gestalt practice, which has always prioritized somatic awareness as a central dimension of therapeutic work [7,48]. The neurobiological framework clarifies why somatic desensitization—one of the most consistently reported bodily manifestations of confluence [3]—may function not only as a symptom but also as a maintenance mechanism: degraded proprioceptive signal quality may prevent salience network engagement, perpetuating the confluent state at the neurobiological level.

This suggests that therapeutic interventions aimed at restoring proprioceptive clarity and somatic self-awareness—grounding exercises, body contact experiments, deliberate attention to muscle tension and bodily sensations—may work not only at the phenomenological level, by increasing awareness, but also at the neurobiological level, by improving the quality of the signal available to the thalamocortical integrative system. We hypothesize that restoring proprioceptive richness may provide the somatosensory network with the input coherence necessary to trigger the activation of the salience network and enable the formation of figures—a claim that requires empirical validation [49]. This hypothesis is consistent with the broader literature on body-based interventions in trauma and dissociation, where somatic approaches have demonstrated efficacy in restoring the clarity of boundaries between self and other in populations characterized by boundary disorders that are neurobiologically related to those proposed here [50,51].

The therapeutic relationship itself can be understood as a regulated organism-environment field in which the therapist, through an attentive and differentiating presence, supports the gradual restoration of the capacity to form figures within the salience network. To this end, the therapist’s somatic and interoceptive awareness—their ability to maintain clear proprioceptive contact with their own bodily state amidst the patient’s confluent field—becomes a therapeutic tool of the first order [52,53]: a therapist who loses somatic self-awareness risks being drawn into the patient’s own pattern of undifferentiated activation, a form of projective identification with both neurobiological and relational dimensions [54,55,56]. Maintaining a grounded, differentiated presence is therefore not merely a clinical skill but may, we hypothesize, function as an act of neurobiological co-regulation.

### 8.2. Gradual Differentiation and Management of Defensive Arousal

The “contact avoidance” dimension of confluence—proposed in Section 7 as potentially related to the amygdala-induced suppression of thalamic signals relevant to salience—has direct implications for the timing and sequence of therapeutic interventions. If self-other differentiation functions as a threat for the confluent client’s nervous system—activating the suppression of the very signals needed for figure formation—then approaches that move too rapidly toward differentiation may paradoxically reinforce confluent functioning by triggering the defensive circuit they aim to dissolve. This suggests the clinical value of graded differentiation: a therapeutic approach that moves gradually toward increasing the distinction between self and other, carefully calibrating the pace of differentiation according to the patient’s current window of affective tolerance [57]. In neurobiological terms, this means working within the activation range where the amygdala-TRN inhibitory circuit is not maximally activated—the range in which the organism can register a homeostatic disturbance without triggering a defensive suppression of salience-relevant signals. Gestalt therapy’s use of the present-moment experiment—small, contained steps of novelty and differentiation within the safety of the therapeutic relationship—can be understood as a clinical operationalization of graded neurobiological regulation: gradually expanding the patient’s tolerance for differentiation without triggering defensive fusion [58].

The thalamocortical model proposed here is not the only available neurobiological account of self-other boundary disruption. Predictive processing and active inference [59], the default mode network [60], alexithymia frameworks [61], and attachment-based models of affective regulation [62] all offer partially overlapping and potentially complementary perspectives on the disruption of self-other differentiation. The thalamocortical framework is presented here as complementary to, rather than exclusive of, these alternatives; its distinctive heuristic value lies in its capacity to account for all three phenomenological dimensions of confluence through a single functional mechanism—the failure of the transition to differentiated figure formation—offering a level of parsimony and structural coherence that invites targeted empirical operationalization. The relationship between these frameworks remains an open and productive question for future theoretical work.

## 9. Conclusions

This article proposes a neurobiological framework for confluence grounded in established neuroscientific literature on thalamic architecture and the salience network. Building on the three-dimensional structure identified by Cioffi et al. (2025) [3]—blurred boundaries, undifferentiation, and avoidance of contact—we propose that dysfunctional confluence can be understood as a chronic disruption of the transition from globally distributed somatosensory activation to differentiated figure formation in the salience network [4,5,6,26,45]. This framework provides Gestalt psychotherapy with an explicit neurobiological account of one of its central clinical constructs—filling a gap recognized by the Gestalt community itself and opening a dialogue with affective neuroscience, which has until now developed in substantial mutual isolation. Clinically, the model suggests that somatic desensitization is not merely a symptom of dysfunctional confluence but may represent a maintenance mechanism: the degradation of the proprioceptive signal could actively prevent the activation of the salience network, perpetuating the confluent state at the neurobiological level. This hypothesis offers a possible mechanistic interpretation of those somatic and grounding interventions already central to the Gestalt tradition, suggesting that their efficacy may operate not only on the phenomenological level but also at the neurobiological level—a hypothesis that remains to be empirically tested. More generally, this work aims to contribute to the growing dialogue between affective neuroscience and humanistic psychotherapy, offering an example of how clinical constructs developed in the experiential domain can be translated into neurobiologically grounded hypotheses amenable to empirical verification.

## 10. Limitations and Future Developments

While this model identifies a putative thalamocortical correlate of dysfunctional confluence, it is unable to specify the mechanism by which this pattern of undifferentiated activation stabilizes in a chronically persistent and context-independent manner—thus distinguishing itself from the temporary and contextually appropriate loosening that characterizes functional confluence. The neurobiological conditions that determine this transition from a flexible and reversible state to a rigid and chronic one remain an open question that future research will need to address. A further limitation concerns the generalizability of the empirical basis from which the model derives: the three-dimensional structure identified by Cioffi et al. (2025) [3] was developed based on contributions from trainers primarily affiliated with Italian institutions associated with FISIG, and the extent to which this structure can be considered representative of Gestalt contexts from different national or institutional traditions remains an open question. On a methodological level, constructs such as contact boundary, undifferentiation, and contact avoidance lack shared operationalizations that would allow direct translation into psychophysiological or neuroimaging variables—a gap that future research will need to address explicitly. Candidate operationalizations for this distinction include interoceptive accuracy measures (e.g., heartbeat detection tasks), proprioceptive sensitivity paradigms (e.g., joint position sense or rubber hand illusion protocols), and resting-state thalamocortical functional connectivity indices derived from fMRI—variables that could in principle differentiate a flexible, context-sensitive loosening of self-other boundaries from a rigid, context-independent fusion. At the clinical level, instruments assessing related constructs—such as the Differentiation of Self Inventory [63,64,65], which measures interpersonal fusion and self-other boundary functioning—could represent a starting point, pending conceptual alignment with the Gestalt construct of confluence and the development of a confluence-specific validated instrument.

No existing study has operationalized confluence in neurobiologically measurable terms [66,67]; therefore, the proposed correspondences between the phenomenological dimensions of confluence and thalamo-cortical dysfunction are theoretical hypotheses, not empirically validated findings. The model functions as a generator of testable hypotheses rather than a synthesis of established findings—a limitation that is simultaneously its main contribution: defining the empirical questions that future research must address. Among the empirical questions that future research could address are: (a) whether individuals with high dysfunctional confluence show altered resting-state functional connectivity within thalamocortical circuits, particularly between thalamic nuclei and the anterior insula, compared to low-confluence controls; (b) whether they display measurable differences in interoceptive and proprioceptive processing, assessable through established psychophysiological paradigms; (c) whether body-oriented Gestalt interventions produce pre-to-post changes in these same neurobiological variables. These represent methodologically feasible lines of inquiry directly derived from the hypotheses advanced here, provided that a validated operationalization of confluence as a measurable variable is first developed.

## Figures and Tables

**Table 1:** The three phenomenological dimensions of confluence (adapted from [3]).

Dimension	Description	Main Clinical Manifestations	Frequency in Contributions (*n* = 8)
Blurred Boundaries	Weakened differentiation between self and environment Indistinct experience of separation between organism and field	Loss of agency Psychotic self-other fusion Fusion states with positive valence (creative flow, early bonding)	7/8
Undifferentiation	Inability to form clear figure-ground distinctions Persistence in the precontact phase	Confusion regarding one’s own needs and feelings Inability to make decisions Erosion of autonomous will	6/8
Contact Avoidance	Active defensive mechanism Withdrawal into protective fusion to elude the vulnerability of authentic differentiation	Somatic desensitization Muscular inhibition Reduced proprioceptive sensitivity	5/8

**Table 2:** Proposed correspondences between the Gestalt contact cycle and the thalamic-salience network system.

Phase of the Contact Cycle	Gestalt Process	Hypothesized Neurobiological Correlate
Sensory Pre-contact	The body constitutes the undifferentiated background from which no figure has yet emerged	Activation of ascending thalamic pathways Generation of a proto-conscious and pre-verbal sense of self
Perceptual Precontact	Emergence of a salient figure from the background Awareness of homeostatic perturbation	Activation of the salience network Amplification of the homeostatically most relevant stimulus against the background of diffuse thalamic activation

Note: The table presents the two pre-contact phases relevant to the proposed model. The complete contact cycle includes additional phases not directly implicated in the neurobiological model of confluence.

**Table 3:** Neurobiological model of confluence: from thalamocortical integration to confluent dysfunction.

Phase of the Model	Gestalt Level	Neurobiological Level	Functional/ Dysfunctional
Distributed Activation	Undifferentiated background Body as pre-reflective seat of the self	Globally distributed somatosensory activation Lemniscal and spinothalamic pathways Protoconscious sense of self	Functional: necessary condition from which every contact cycle emerges
Filtering and Amplification	Progressive emergence of the relevant homeostatic perturbation	TRN selectively filters signals ARAS modulates baseline arousal Amygdala weighs emotional relevance	Functional: selects what will become a figure
Figure Formation	Background figure transition Initiation of the contact cycle	Salience network activation threshold reached Anterior insula and dACC differentiate the figure	Functional: the Gestalt forms Contact becomes possible
Transition Failure by Deficit	Blurred boundaries/undifferentiation: figure does not emerge Cycle blocked at its origin	Insufficient spatiotemporal coherence of the ascending signal Salience Network not engaged	Dysfunctional: confluence as a chronic state independent of context
Transition Failure by Suppression	Contact avoidance: differentiation itself activates defensive fusion	Amygdala→TRN circuit actively suppresses relevant signals before the figure formation threshold	Dysfunctional: confluence as an active defensive mechanism

## Data Availability

No new data were created or analyzed in this study. Data sharing is available from the corresponding author upon reasonable request.
